# A Cluster Set Protocol in the Half Squat Exercise Reduces Mechanical Fatigue and Lactate Concentrations in Comparison with a Traditional Set Configuration

**DOI:** 10.3390/sports8040045

**Published:** 2020-04-04

**Authors:** Daniel Varela-Olalla, Alejandro Romero-Caballero, Juan Del Campo-Vecino, Carlos Balsalobre-Fernández

**Affiliations:** Department of Physical Education, Sport and Human Movement, Universidad Autónoma de Madrid, 28049 Madrid, Spain; alexromero9c@gmail.com (A.R.-C.); jdelcampovecino@yahoo.es (J.D.C.-V.); info@carlos-balsalobre.com (C.B.-F.)

**Keywords:** resistance training, fatigue, performance, biomechanics, exercise prescription, velocity-based training

## Abstract

Splitting sets into clusters has been shown to maintain performance during resistance training. This study compared the acute fatigue produced by a traditional (TSC) versus a cluster (CSC) set configuration in the smith machine half squat exercise. Fifteen males performed a single bout of TSC and CSC separated by 72–96 h. In the TSC, participants performed as many repetitions as possible until reaching a 20% drop in barbell velocity (MPV), while in the CSC, they performed the same number of repetitions with 15 seconds inter-repetition rest. Effects of both protocols in MPV, countermovement jump height (CMJ), and blood lactate (BLa) were measured. Significant differences between protocols were found for MPV of the last repetition (0.4 vs 0.5 m/s TSC and CSC) and BLa (6.8 mmol/L vs 3.2 mmol/L TSC and CSC). Significant drop of velocity from the first to the last repetition of the set (19.9%), decrease in CMJ height (35.4 vs 32.6 cm), and increase in BLa (2.1 vs 6.8 mmol/L) pre–post-exercise was observed just for the TSC protocol. The results of the present study showed that CSC reduces the lactate response and mechanical fatigue produced by a single set on the half squat exercise in comparison with TSC.

## 1. Introduction

Optimization of training interventions is crucial to elicit the desired neuromuscular adaptations during resistance training [1]; thus, strength and performance can be improved by proper manipulation of different training variables such as exercise, sets, repetitions or rest period [2].

It has been widely argued in the scientist literature that training to muscular failure (i.e., performing the maximum number of repetitions possible with a given relative load) is unnecessary and probably detrimental to maximizing athletic performance [3,4]. Velocity loss (VL) has been recently proposed as a precise and non-invasive variable to monitor the actual degree or level of effort incurred during resistance training [5], a very high relationship having been found between the relative velocity loss in a training set and the percentage of performed repetitions with respect to the maximum possible number that can be completed in the bench press and pull-up exercises [6,7]. For this reason, training methods that reduce the amount of induced fatigue and allow maintenance of maximum velocity in every repetition during resistance training have been shown to optimize strength and performance gains [8,9].

Inter-repetition rest (IRR) is a variable that is gaining popularity among practitioners and researchers and refers to the cluster set methodology which includes short rest intervals between repetitions or block of repetitions during a set, in contrast to the traditional set where all repetitions are performed consecutively [10]. The increasing interest in this type of training is due to the fact that some studies have reported greater maintenance and improvements of movement velocity and power output during a set [11,12,13], reduced neuromuscular fatigue [13], greater training volumes without decreasing repetition performance [14], and also grater loads being lifted with similar velocity loss [10]. 

The literature analyzing chronic responses to cluster training compared to traditional protocols seems controversial. Some studies show greater improvements in barbell velocity and power output [11,15] and muscular strength [15], while others have not found differences in the improvements in strength, jump height, and mechanical variables between protocols [16,17,18].

In general, cluster protocols seem to acutely reduce fatigue and help to maintain neuromuscular performance relative to kinetic and kinematic variables compared to traditional protocols with pre-established sets per repetition schemes if the same amount of work is done. However, there is little evidence comparing cluster training protocols with velocity-based protocols and not preset repetitions and percentages.

Thus, the objective of the present work was to compare the acute effects of a single bout of resistance exercise on metabolic (lactate) and mechanical (jump height and barbell velocity) indexes of fatigue for the lower limbs of recreationally trained subjects, performing the same number of repetitions in a traditional set configuration (TSC) versus a cluster set configuration (CSC). We hypothesized that CSC would further reduce the amount of fatigue accumulated in a single bout of resistance training compared to a TSC even when a moderate velocity loss of 20% fatigue is achieved during TSC.

## 2. Materials and Methods

### 2.1. Subjects

Fifteen healthy recreationally active male subjects (age = 23.0 ± 2.4 years; height = 175.0 ± 6.0 cm; body mass = 73.1 ± 8.2 kg; 1 repetition maximum (RM) = 99.7 ± 13.1kg; 1RM mean propulsive velocity (MPV) = 0.3 ± 0.1 m/s) volunteered to take part in this study. None of the subjects had physical limitations, health problems or injuries at the time of the test. None of the participants were taking drugs, medications, supplements or other substances that could alter their physical performance. The informed consent of each participant was obtained. The study protocol was approved by the Autonomous University of Madrid Ethics Committee, according to the Declaration of Helsinki for Human Experimentation.

### 2.2. Experimental Design

The investigation was carried out on three different days separated by 72–96 hours. All testing sessions were carried out at the same time of the day for all subjects. On the first day, the participants performed an incremental loading test until 1RM for the half squat exercise with loads that were progressively increased from 20 kg with 10 kg increments in each set until a MPV of ≈ 0.3 m/s was reached (with 3 min of rest between sets); individual load–velocity profiles for each subject were obtained. On the second day, the participants made a traditional set configuration (TSC) with their individual 0.5 m/s load, until losing 20% of MPV. Barbell MPV at the beginning and at the end of the set, and the number of repetitions made was noted. On the third day, the participants made a cluster set configuration (CSC) (with a 15 s rest between repetitions) with the 0.5 m/s load until the same number of repetitions as the previous day. Days two and three were not randomized, since it was first necessary to know the number of repetitions performed during TSC to be able to match them during CSC. 

Barbell MPV at the beginning and at the end of the set was also noted. The participants were instructed to perform the propulsive phase with the maximum possible velocity in each repetition. All tests were carried out in a Smith machine. Before and after the tests on days two and three, lactate values were taken, and a countermovement jump (CMJ) was performed. A total of 24 h after each measurement, the participants completed a single-item questionnaire on delayed onset muscle soreness (DOMS). No nutritional control of the diet, except for the use of drugs, medications, supplements or other specific substances that could affect performance, was carried out.

### 2.3. Testing Procedures 

#### 2.3.1. Load–Velocity Profile Determination

Before the commencement of the test, participants performed a standardized warm-up consisting of 5 min of joint mobility, followed by 2 sets of 10 and 8 repetitions (interspaced with 2 min rest) with an external load of 20 and 30 kg, respectively. After warming up, the individual load–velocity relationship was determined by means of a standard incremental loading test performed in a Smith machine [19]. The initial external load of the incremental loading test was set at 20 kg. Thereafter, the load was progressively increased in 10 kg increments until the MPV was ≈ 0.3 m/s. From that moment, the load was progressively increased in steps of 5 to 2.5 kg until the subject 1RM strength was achieved.

#### 2.3.2. Resistance Exercise Tests

Before the commencement of the tests, participants performed a standardized warm-up consisting of 5 min of joint mobility, followed by 4 sets of 10, 5, 3, and 2 repetitions (interspaced with 3 min rest) with external loads of 20, 30, 50, and 70 kg, respectively. All repetitions of the warm-up were monitored with the linear transducer to estimate the 0.5 m/s load, based on the individual load–velocity profile obtained during the incremental test, with which each participant performed the test. Immediately before both tests, lactate values and CMJ height were measured in that specific order.

Traditional set configuration consisted on a straight set (all repetitions were performed without any rest in between, as is usually done during traditional resistance training) of half squat until a velocity loss of 20% with respect to the fastest repetition of the set. At the end of the test, the total number of repetitions was noted in order to achieve the same number of repetitions in the cluster set configuration test. Immediately before the end of the test, lactate values and CMJ height were measured again in that specific order.

Cluster set configuration consisted in a set of half squat in which subjects had to perform the same number of repetitions they did during the traditional set configuration set with 15 s rest between repetitions. Immediately before the end of the test, velocity loss between the fastest and the last repetition was noted, and lactate values and CMJ height were measured again in that specific order.

### 2.4. Instruments

The Speed4Lift linear transducer was used for recording barbell MPV. The linear transducer cable was attached to the barbell aligned with the vertical axis, following the criterion described by the manufacturer. MPV values in m/s were recorded for each repetition with a sampling frequency of 1000 Hz. This device has been validated recently [20].

The MyJump 2 iOS app was used to measure jump height by calculating the flight time of the CMJ by identifying the takeoff and the landing frames of the video and then using the equation described by Bosco, Luthanen, and Komi [21] to transform it into a jump height [22]. The app was installed on an iPhone 8, which includes a 240 fps high-speed camera, at a quality of 1080 pixels. 

A Lactate Pro Portable Analyzer (Arkray, Kyoto, Japan) was used to measure lactate values. Blood lactate samples were taken before the start of each set (but after the warm-up) and then 3 min after the last repetition. Capillary blood samples (5 µL) were collected from the index finger of the left hand and immediately analyzed using the portable analyzer. Each day, before the start of the measurements, the analyzer was calibrated following the manufacturer’s recommendations.

A single-item questionnaire on DOMS was provided to the subject 24 h after each test, in which they had to answer the question: "How intense the pain is in your lower limbs?”, 0 being “no pain” and 10 “unbearable pain”, on a Likert scale.

### 2.5. Statistical Analysis

Descriptive data are presented as means, standard deviations (SD), and coefficients of variation (CV). The distribution of the variables was analyzed using the Kolmogorov–Smirnov test, and homogeneity of variances was tested via Levene´s test. Differences between the traditional set and cluster set configurations for the main variables analyzed were tested using the independent sample T test; additionally, the paired sample T test was used to analyze differences for the velocity of the last repetition (MPV_last_) against the velocity of the best repetition (MPV_best_) in both protocols. Differences between the pre- and post-test for CMJ height and lactate values obtained in each protocol were tested using the paired sample T test. The level of significance was established at *p* ≤ 0.05. Statistical analyses were performed using the software package IBM SPSS Version 23.0 (SPSS Inc., Chicago, IL, USA).

## 3. Results

The data obtained for each variable during both testing protocols are presented in Table 1. Significant differences were found between protocols for MPV_last_ showing lower values for TSC and for velocity loss, lactate_post_ and difflact showing higher values for TSC; additionally, after comparing MPV_last_ against MPV_best_, statistically significant differences were found only for TSC.

**Figure 1:**
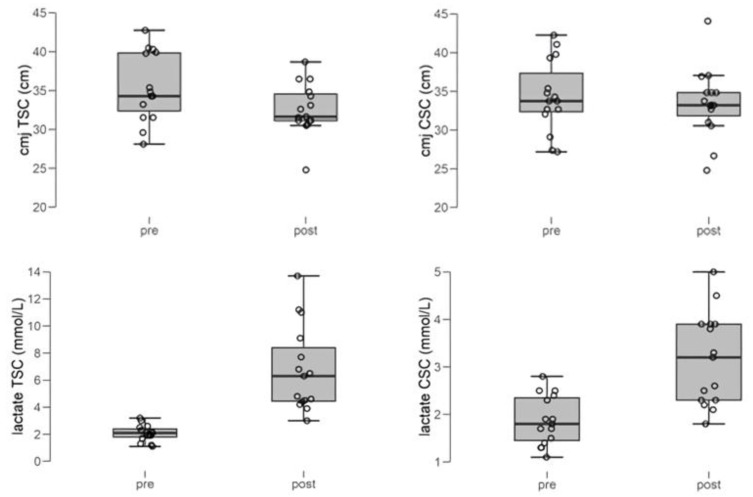
Pre–post differences for CMJ height and lactate values in the TSC and CSC groups. Significant (*p* < 0.05) differences for CMJ height were found only for TSC. Significant (*p* < 0.05) differences for lactate were found for both protocols. Note the differences in the values of the Y axis between TSC and CSC for the comparisons of lactate concentrations.

CMJ height showed statistically significant reductions only after TSC, whilst lactate values were significantly higher after both protocols in comparison to the baseline (Figure 1).

## 4. Discussion

The objective of the present study was to compare the acute response of fatigue in recreationally-trained subjects after performing the same number of repetitions in the half squat exercise using a TSC versus a CSC. The main findings of our work are that the CSC protocol produces a lower reduction in MV over the set and lower levels of lactate concentrations and lower reductions in CMJ height after the set compared to the TSC protocol. The abovementioned results confirm our hypothesis that fatigue is further reduced with CSC protocols versus with TSC protocols even when velocity loss is limited to a moderate degree.

From the results listed above, it can be inferred that the cluster set is an interesting option for reducing fatigue based on significant differences between protocols for CMJ height and velocity loss, which have been found to be two reliable mechanical indicators of fatigue [5]. On the other hand, lactate concentrations showed significant increments after both protocols, indicating disparity between metabolic and mechanical indicators of fatigue in this regard; however, the values obtained after TSC were significantly higher than those in CSC (Table 1). This can be explained by the fact that lactate can be considered as a metabolic link between glycolytic and aerobic metabolism, being produced during glycolysis and used as a substrate during mitochondrial respiration [23]. This could imply that lactate goes over basal values the moment the glycolytic metabolism is “activated” during exercise, and it only reaches a plateau or a decline if mitochondrial capacity can compensate for energetic cellular needs (which is difficult during resistance exercise) as proposed elsewhere [24].

Evidence has also shown that lactate concentrations after resistance exercise did not differ between protocols using different relative loads and rep schemes [25], and also increases in lactate concentrations were found after just one maximal vertical jump as a result of the activation of the glycolytic metabolism on the very onset of exercise [26]. Based on the above, glycolytic demands of resistance training explain the significant increases in lactate concentrations observed after both protocols, whereas the significant lower values obtained after the CSC protocol could be due to phospocreatine resynthesis (PCr) during IRR (50% of PCr can be resynthesized in the first 30 s of rest [27]) reducing glycolytic needs during the set.

Our results are in line with those of previous studies that have shown lower velocity loss and lactate concentrations after four different resistance exercise protocols (varying volume and relative load) using 10 and 20 s IRR compared to traditional training sets of full squats performed in a smith machine [13], and greater ability of the subjects to maintain mean velocity and peak power when performing cluster sets of 30 s compared to traditional sets in the free-weight back squat exercise [12]. In addition, lower velocity loss and lactate concentrations were also found when comparing two strength- and hypertrophy-oriented TSC protocols against two different strength-oriented CSC protocols with different relative loads and 25 s IRR for the back squat exercise [17]. Similar to the above findings, CSC protocols analyzed in upper-body movements have also found lower drops in velocity and lactate concentrations for the bench press exercise when performing four sets of five repetitions with 30 s IRR compared to four sets of five repetitions using a TSC without IRR [18]. Finally, other authors have found that cluster set structures of twelve repetitions performing 30 s of intra-set rest every two or four repetitions allowed greater loads, total work, and time under tension without affecting peak power and velocity compared to the traditional set structure of twelve straight repetitions [10], also indicating that cluster sets are a viable option to reduce neuromuscular fatigue for the same load or to increase the external load for the same degree of fatigue.

The main limitations of the present work are the utilization of the half squat exercise in a smith machine which points out the necessity of being cautious when extrapolating results to other exercises such as the full squat, since different responses have been found between lower-body and upper-body movements when performing cluster training [15], and also to exercises performed with a barbell (free-weight exercises) and not in a smith machine. Another limitation is the level of experience and strength of the subjects; we recruited healthy active male subjects with a relative strength around 1.36 kg/kg body mass; thus, experience with lower (i.e., <1 kg/kg body mass) and higher (i.e., >1.5 kg/kg body mass) levels of strength should be studied implementing similar protocols. In addition, no female subjects participated in the study, and comparing response to CSC protocols between males and females could be an important and interesting line for future research. Lastly, we only analyzed the difference for a velocity loss of 20%, and differences in CSC protocols equaling various levels of this variable should be investigated in the future.

## 5. Conclusions

In summary, the results of the present study suggest that adding an IRR of 15 s between repetitions (CSC) allows reducing even more (i) the velocity loss during and at the end of the sets and (ii) the CMJ height loss, compared with TSC with a moderate velocity loss when the same number of repetitions is performed in both protocols. Despite significant differences between lactate values pre- vs. post-training in both protocols, our results showed that the values obtained after CSC are significantly lower than those obtained after TSC accompanying the reduction in mechanical fatigue with the use of cluster methodology.

The main practical application of this research is that when maintaining neuromuscular performance and minimizing fatigue is relevant in the training program, CSC seems a better strategy than TSC. Despite these findings, some limitations need to be addressed, as the subjects were recreationally-active males, so our results should not be extrapolated to elite athletes, and it could be interesting to replicate this work with this population and also with females. Another important consideration should be made in regard to the material used, as a smith machine was used for the tests and results could be different using free-weight exercises.

## Figures and Tables

**Table 1:** Descriptive variables obtained during traditional (TSC) and cluster set configuration (CSC) protocols.

Variable	Testing Protocol			
	TSC		CSC	
	Mean ± SD (Range)	CV (95%CI)	Mean ± SD (Range)	CV (95%CI)
Load (kg)	62.8 ± 9.2 (50–82.5)	14.6 (57.7–67.9)	61.2 ± 11.5 (50–90)	18.8 (54.8–67.5)
MPV_best_ (m/s)	0.5 ± 0.03 (0.45–0.55)	6 (0.47–0.51)	0.5 ± 0.03 (0.45–0.53)	5.32 (0.48–0.49)
MPV_last_ (m/s) *	0.4 ± 0.02 (0.36–0.45) ^‡^	6.1 (0.38–0.41)	0.5 ± 0.03 (0.41–0.53)	6.7 (0.46–0.49)
Velocity Loss (%) *	19.9 ± 3.2 (14.9–26)	16.2 (18.1–21.7)	2.4 ± 4.8 (−4.44–10.6)	197.9 (−0.23–5.07)
CMJ_pre_ (cm)	35.4 ± 4.4 (28.1–42.8)	12.4 (32.9–37.8)	34.4 ± 4.7 (27.2–42.2)	13.5 (31.8–36.9)
CMJ_post_ (cm)	32.6 ± 3.3 (24.8–38.7)	10.2 (30.8–34.4)	33.4 ± 4.5 (24.8–44.1)	13.4 (31–35.9)
DiffCMJ (cm)	2.8 ± 2.8 (−1.94–9.29)	103.3 (1.18–4.33)	0.9 ± 2.9 (−2.65–7.33)	311.3 (−0.67–2.52)
Lactate_pre_ (mmol/L)	2.1 ± 0.6 (1.1–3.2)	29.3 (1.74–2.41)	1.9 ± 0.5 (1.1–2.8)	27.8 (1.59–2.16)
Lactate_post_ *(mmol/L)	6.8 ± 3.2 (3–13.7)	46.7 (5.03–8.54)	3.2 ± 1.0 (1.8–5)	31 (2.61–3.69)
Difflact * (mmol/L)	4.7 ± 3.1 (−10.5–0)	66.6 (−6.44–(−2.47))	1.3 ± 0.7 (−2.2–0.6)	57.7 (−1.69–(−0.87))
DOMS	3.2 ± 2.5 (0–8)	76.7(1.84–4.56)	1.9 ± 2.1 (0–7)	109.7 (0.76–3.11)

SD: standard deviation; CV: coefficient of variation; CI: confident intervals; MPV_best_: mean propulsive velocity of fastest repetition; MPV_last_: mean propulsive velocity of last repetition; DiffCMJ: difference between countermovement jump height (CMJ) pre–post-test; Difflactate: difference between lactate pre–post-test; * significant differences between protocols (*p* < 0.001); ^‡^ significant differences for MPV_last_ against MPV_best_ for the same protocol (*p* < 0.001).

## References

[1] Kraemer W.J., Ratamess N.A. (2004). Fundamentals of resistance training: Progression and exercise prescription. Med. Sci. Sports Exerc..

[2] Bird S., Tarpenning K., Marino F. (2005). Designing Resistance Training Programmes to Enhance Muscular Fitness. Sports Med..

[3] Davies T., Orr R., Halaki M., Hackett D. (2016). Erratum to: Effect of training leading to repetition failure on muscular strength: A systematic review and meta-analysis. Sports Med..

[4] Izquierdo M., Ibañez J., González-Badillo J.J., Häkkinen K., Ratamess N.A., Kraemer W.J., Gorostiaga E.M. (2006). Differential effects of strength training leading to failure versus not to failure on hormonal responses, strength, and muscle power gains. J. Appl. Physiol..

[5] Sánchez-Medina L., González-Badillo J.J. (2011). Velocity loss as an indicator of neuromuscular fatigue during resistance training. Med. Sci. Sports Exerc..

[6] Gonzalez-Badillo J.J., Yanez-Garcia J.M., Mora-Custodio R., Rodriguez-Rosell D. (2017). Velocity loss as a variable for monitoring resistance exercise. Int. J. Sports Med..

[7] Sanchez-Moreno M., Rodriguez-Rosell D., Pareja-Blanco F., Mora-Custodio R., Gonzalez-Badillo J.J. (2017). Movement velocity as indicator of relative intensity and level of effort attained during the set in pull-up exercise. Int. J. Sports Physiol. Perform..

[8] Padulo J., Mignogna P., Mignardi S., Tonni F., D’Ottavio S. (2012). Effect of Different Pushing Speeds on Bench Press. Int. J. Sports Med..

[9] Pareja-Blanco F., Rodríguez-Rosell D., Sánchez-Medina L., Sanchis-Moysi J., Dorado C., Mora-Custodio R., González-Badillo J.J. (2017). Effects of velocity loss during resistance training on athletic performance, strength gains and muscle adaptations. Scand. J. Med. Sci. Sports.

[10] Tufano J., Brown L., Haff G. (2017). Theoretical and Practical Aspects of Different Cluster Set Structures. J. Strength Cond. Res..

[11] Morales-Artacho A., Padial P., García-Ramos A., Pérez-Castilla A., Feriche B. (2018). Influence of a Cluster Set Configuration on the Adaptations to Short-Term Power Training. J. Strength Cond. Res..

[12] Wagle J., Taber C., Carroll K., Cunanan A., Sams M., Wetmore A., Stone M.H. (2018). Repetition-to-Repetition Differences Using Cluster and Accentuated Eccentric Loading in the Back Squat. Sports.

[13] Mora-Custodio R., Rodríguez-Rosell D., Yáñez-Garcí J., Sánchez-Moreno M., Pareja-Blanco F., González-Badillo J. (2018). Effect of different inter-repetition rest intervals across four load intensities on velocity loss and blood lactate concentration during full squat exercise. J. Sports Sci..

[14] Tufano J., Halaj M., Kampmiller T., Novosad A., Buzgo G. (2018). Cluster sets vs. traditional sets: Levelling out the playing field using a power-based threshold. PLoS ONE.

[15] Oliver J., Jagim A., Sanchez A., Mardock M., Kelly K., Meredith H., Fluckey J.D. (2013). Greater Gains in Strength and Power with Intraset Rest Intervals in Hypertrophic Training. J. Strength Cond Res..

[16] Iglesias-Soler E., Mayo X., Río-Rodríguez D., Carballeira E., Fariñas J., Fernández-Del-Olmo M. (2016). Inter-repetition rest training and traditional set configuration produce similar strength gains without cortical adaptations. J. Sports Sci..

[17] Nicholson G., Ispoglou T., Bissas A. (2016). The impact of repetition mechanics on the adaptations resulting from strength-, hypertrophy- and cluster-type resistance training. Eur. J. Appl. Physiol..

[18] Davies T., Halaki M., Orr R., Helms E., Hackett D. (2019). Changes in Bench Press Velocity and Power After 8 Weeks of High-Load Cluster- or Traditional-Set Structures. J. Strength Cond. Res..

[19] García-Ramos A., Pestaña-Melero F.L., Pérez-Castilla A., Rojas F.J., Gregory Haff G. (2018). Mean velocity vs. mean propulsive velocity vs. peak velocity: Which variable determines bench press relative load with higher reliability?. J. Strength Cond. Res..

[20] Pérez-Castilla A., Piepoli A., Delgado-García G., Garrido-Blanca G., García-Ramos A. (2019). Reliability and Concurrent Validity of Seven Commercially Available Devices for the Assessment of Movement Velocity at Different Intensities during the Bench Press. J. Strength Cond. Res..

[21] Bosco C., Luhtanen P., Komi P.V. (1983). A simple method for measurement of mechanical power in jumping. Eur. J. Appl. Physiol. Occup. Physiol..

[22] Balsalobre-Fernández C., Glaister M., Lockey R.A. (2015). The validity and reliability of an iPhone app for measuring vertical jump performance. J. Sports Sci..

[23] Brooks G.A. (2018). The Science and Translation of Lactate Shuttle Theory. Cell Metab..

[24] Tesch P.A., Colliander E.B., Kaiser P. (1986). Muscle metabolism during intense, heavy-resistance exercise. Eur. J. Appl. Physiol. Occup. Physiol..

[25] Nitzsche N., Baumgartel L., Weigert M., Neuendorf T., Frholich M., Schulz H. (2017). Acute effects of three resistance exercise programs on energy metabolism. Int. J. Sport Sci..

[26] Chamari K., Ahmaidi S., Blum J.Y., Hue O., Temfemo A., Hertogh C., Mercier J. (2001). Venous blood lactate increase after vertical jumping in volleyball athletes. Eur. J. Appl. Physiol..

[27] Maughan R., Gleeson M. (2010). The Biochemical Basis of Sports Performance.

